# Artificial Feeding of Infants

**Published:** 1911-06-15

**Authors:** H. F. Slifer


### ARTIFICIAL FEEDING OF INFANTS.

BY H. F. SLIFER, M.D.

Statistics from the registered deaths in England, compiled by Dr. Hugh R. Jones (*Brit. Med. Jour.*), show that 42 per cent. of infant deaths may be referred to digestive disorders. Infant mortality in Norway and Sweden, where almost every child is nursed by its own mother, is but 10 per cent. In Wurtemburg the mortality of breast-fed children is 13.5 per cent., of artificially-fed 42.7 per cent. In Munich, of the total number of deaths, 15 per cent. are of breast-fed children and 85 per cent. of artifically-fed. In lower Bavaria, where maternal nursing is the exception, the mortality is 50 per cent.

An examination of American statistics prove the applicability of the above statement to this country. If death-rate has any claim to direct professional attention, it appears to the writer that this a is strong appeal to enlist our interest in behalf of the dying children.

The past teaches us that the evil of artificial feeding of infants is increasing as civilization advances. We should recognize the fact that a cause or a combination of causes that destroys 20 to 50 per cent. of the infant population, is a question of vital importance, involving the welfare of the State as well as the individual. At no time of life is food so important, and nutrition so much desired, as during the constructive period of early infancy. At this stage, the physical foundation for the future structure is formed. If nutrition is impaired at this age, the body will be enfeebled, the organs defective, and consequently growth and development will be imperfect, with no power to resist the baneful influence of disease. The food and methods of feeding are responsible in a great degree for this condition. It is the function of the physician to know the nature of the food, how produced and how presented to the market, and in what manner it is given to his helpless patient. Food for the infant should be a fluid containing all the elements necessary for nutrition; sterile, faintly alkaline, at a temperature of 100° F., and furnished in proper amount so as not to distend the stomach beyond its physiological limit.

Experience teaches that the best substitute for mother's milk is cows'milk, modified and adapted to the age and peculiarity of the child. Cow's milk differs from human milk in several important particulars. According to recent analyses published by Dr. Rotch, of Boston, the constituents of the two are as follows:

| | Human | Cow |
|---|---|---|
| Reaction | Alkaline. | Acid. |
| Bacteria | Absent. | Present. |
| Specific gravity | 1031 | 1032 |
| Fats | 4.00 | 4.00 |
| Sugar | 7.00 | 4.50 |
| Proteids | 1.50 | 4.00 |
| Ash | .15 | .70 |
| Total Solids | 12.65 | 13.20 |

The reaction of cows' milk as served to the public, is acid owing to the action of bacteria on milk sugar. Bacteriologists have found that souring of milk is due to the presence of a hundred different kinds of micro-organisms. The addition of an alkali is, therefore, indicated to counteract this acidity. Lime water, soda bicarbonate, and, if constipation is present, soda salicylate, soda phosphate or magnesia are preferable. The amount should be sufficient to render the milk alkaline. "It has been demonstrated that about two hundred different kinds of bacteria exist in market milk. That, as ordinarily drawn from the cow, the milk, by the time it is in the pail, will contain anywhere from 1,500 to 6,000,000 bacteria per cubic inch." Many of these, fortunately are harmless, but of no advantage to the milk for infant-feeding, and favor fermentation. Other forms are pathogenic and are the direct cause of many gastro-intestinal disorders in children. Some bacteria are important to the butter-maker, since they are the agents by which cream is ripened and the flavor of butter produced. Also, the flavor of the different varieties of cheese is the result of bacterial action. There is again another class of bacteria whose effect is deleterious. They produce decomposition products, sometimes bitter, sometimes putrefied, and again decidedly poisonous. Milk thus contaminated must be pasteurized (167 degrees F.) or sterilized (212 degrees F.) to arrest bacterial growth.

It is possible, by extreme precaution, to have milk nearly free from bacteria, postponing fermentation for an indefinite time, and thus removing one of the objectionable features of artificial feeding. Sterilization would be unnecessary and the milk would be in its natural state. The percentage of fats in human and cow milk are about equal. A reduction in the per cent. of fat is not objectionable so long as the child is properly nourished. If, however, more fat is indicated and constipation is present, top milk or cream should be added. Cow's milk is poor in sugar. This being an important element for the production of force and readily digested, it should be supplied in the form of milk or cane sugar. The proteids are abundant in cow's milk, which renders it difficult of digestion by the infant. It must, therefore, be diluted, so as to agree with the percentage of proteids in human milk.

To be successful in the practice of infant-feeding, the milk must be of the best quality, taken from a mixed herd, of ordinary breed, fed upon the most approved rations, and cared for in the best possible manner. The milk should be taken from the cow as free from contamination with dirt and bacteria as possible, then at once aerated and artifically cooled and temperature maintained at a point not above (50° F.,) until it reaches the consumer. If milk is strictly fresh and pure and properly cared for, it is not necessary to sterilize, but simply to heat to the temperature of the body (100° F.) when served to the child.

The mode of feeding should be simple. Infants, under one year of age, should feed exclusively from a bottle. The bottle should be plain, with a short neck, and of a capacity adapted to the age of the child. The bottle may be graduated, so that the proper amount may be given at each feeding. The nipple should be plain rubber with a small aperture. The child while nursing should repose in a comfortable position, and be attended by an intelligent person who will remove the bottle as soon as the supply of milk is exhausted. The bottle should be immediately cleaned and placed in water, or water with the addition of soda or borax, until the next feeding.

The quantity prescribed at each feeding must not exceed the capacity of the stomach. Dr. L. Emmett Holt's observations show that at birth the average capacity is one ounce, and a gain of one ounce per month up to six months, and for every subsequent month one-half ounce up to one year; hence at this time the stomach is capable of holding nine ounces.

In regulating the amount of milk, we should not ignore the fact that individuals differ in their physiological demand.

The following index has been of service to me in adjusting the proper quantity:

| Age. | Interval. | Amount. |
|---|---|---|
| 1 month. | Every 2 hours during the day. | 1 oz. |
| 2 months. | Every 2 hours during the day. | 2 oz. |
| 3 months. | Every 2 hours during the day. | 3 oz. |
| 4 months. | Every 2½ hours during the day. | 4 oz. |
| 5 months. | Every 2½ hours during the day. | 5 oz. |
| 6 months. | Every 2½ hours during the day. | 6 oz. |
| 12 months. | Every 3 hours during the day. | 6 to 8 oz. |

The fact that cow's milk is rich in proteids, fats and salts, renders it of practical importance to modify it by dilution, so as to resemble human milk. The water employed for dilution must be boiled to destroy organic matter which it may contain. Water not only acts as a diluent, but alters the physical condition of the casein and renders it more digestible by preventing the formation of large coagula during peptogenic digestion. The degree of dilution necessarily depends upon the composition of the milk, the age and condition of the child. The following is my rule for dilution:

| | |
|---|---|
| 1 month, | 1 part milk to 3 parts water. |
| 2 months, | 1 part milk to 2 parts water. |
| 3 months, | 2 parts milk to 2 parts water. |
| 6 months, | 3 parts milk to 2 parts water. |
| 9 months, | 3 parts milk to 1 part water. |
| 12 months, | 6 parts milk to 1 part water. |

If all the elements of the milk are properly digested, the quantity of water is gradually reduced to the proportion of the succeeding month. The strength of the milk is adjusted to the digestive capacity of the child. Milk that is properly diluted so as to give the correct per cent. of proteids, requires the addition of such ingredients as are deficient.

Sugar should be added to the amount of ten or fifteen grains to the ounce. If a good quality of milk is used, it is seldom necessary to add cream.

To be successful in infant feeding, specific rules must be observed. Uniformity in dilution and scrupulous care in the preparation of milk, is indispensible. In my practice, I use printed rules, that I give to the attendant, and insist on strict compliance with them, and thus avoid error and neglect.—*Medical and Surgical Reporter.*

